# Chaotic Color Image Encryption Based on Eight-Base DNA-Level Permutation and Diffusion

**DOI:** 10.3390/e25091268

**Published:** 2023-08-28

**Authors:** Wei Fan, Taiyong Li, Jianan Wu, Jiang Wu

**Affiliations:** School of Computing and Artificial Intelligence, Southwestern University of Finance and Economics, Chengdu 611130, China

**Keywords:** color image encryption, chaotic system, permutation, diffusion, eight-base DNA

## Abstract

Images, as a crucial information carrier in the era of big data, are constantly generated, stored, and transmitted. Determining how to guarantee the security of images is a hot topic in the information security community. Image encryption is a simple and direct approach for this purpose. In order to cope with this issue, we propose a novel scheme based on eight-base DNA-level permutation and diffusion, termed as EDPD, for color image encryption in this paper. The proposed EDPD integrates secure hash algorithm-512 (SHA-512), a four-dimensional hyperchaotic system, and eight-base DNA-level permutation and diffusion that conducts on one-dimensional sequences and three-dimensional cubes. To be more specific, the EDPD has four main stages. First, four initial values for the proposed chaotic system are generated from plaintext color images using SHA-512, and a four-dimensional hyperchaotic system is constructed using the initial values and control parameters. Second, a hyperchaotic sequence is generated from the four-dimensional hyperchaotic system for consequent encryption operations. Third, multiple permutation and diffusion operations are conducted on different dimensions with dynamic eight-base DNA-level encoding and algebraic operation rules determined via the hyperchaotic sequence. Finally, DNA decoding is performed in order to obtain the cipher images. Experimental results from some common testing images verify that the EDPD has excellent performance in color image encryption and can resist various attacks.

## 1. Introduction

Nowadays, with the rapid development of Internet technology and the widespread use of intelligent devices, the network has been integrated into people’s daily lives, and the corresponding information security issues have attracted more attention. In the era of big data, a huge amount of information is transmitted on the network every moment. As an effective and universal information carrier, images’ security is of particular interest [1]. Illegal access to images by unauthorized users is a major concern, and the encryption of images is an effective means of protecting image information. However, since images feature bulky data, high redundancy, and strong correlation between pixels, traditional encryption algorithms such as the data encryption standard (DES), advanced encryption standard (AES), and international data encryption algorithm (IDEA) are not suitable for image encryption. In order to deal with this problem, the popular technique of chaos-based image encryption has been widely discussed and applied by researchers [2,3,4]. Numerous routes have been taken in the design and innovation of current chaotic image encryption schemes, such as introducing and using S-box [5,6,7], Latin square  [8,9,10], multiple data-level encryption operations [11,12,13], and different chaotic systems [14,15,16]. Additionally, chaotic systems can also take on various forms, such as continuous systems [17,18], fractional-order systems [19,20], complex systems [21,22], and discrete systems [23,24,25]. They have advantages such as pseudorandomness, synchronization, ergodicity, and extreme sensitivity to initial values and parameters, and some of these properties are beneficial for image encryption [26]. In recent years, various chaotic image encryption approaches have been demonstrated to be very effective [27,28,29].

Diffusion is used to change the values of pixels in images, and permutation is one of the confusion methods that can be dedicated to disrupting the positions of pixels. These are among the most commonly used image encryption operations, and it is also possible to handle both diffusion and permutation jointly. Researchers usually design image encryption algorithms based on these operations and chaotic systems, mainly conducting diffusion and/or permutation according to the pseudorandom sequences generated from chaotic systems to ensure excellent security and robustness [10,30,31]. Valandar et al. proposed a fast color image encryption method based on a three-dimensional chaotic map and used the strategy of dividing the image to perform permutation and diffusion [32]. Li et al. used a skew tent system and a Rucklidge system to design an efficient and secure color image encryption scheme based on bit-level permutation, and the algorithm processed three color components simultaneously and considered the correlation between them [33]. Liu et al. presented a joint permutation and diffusion color image encryption algorithm based on a Hopfield chaotic neural network [34]. Ref. [35] employed a four-dimensional chaotic system to generate a pseudorandom sequence for consequent operations and then conducted image encryption via multiple-bit permutation and diffusion according to the chaotic sequence. Hua et al. designed a color image encryption method based on cross-plane permutation and non-sequential diffusion by constructing a two-dimensional logistic tent modular map [36]. Ge et al. proposed a novel diffusion scheme for hyperchaotic image encryption, following the “divide and conquer” strategy [37,38]. Each of these techniques performed successfully in resisting different types of attacks. Intuitively, image encryption operations are not limited to the pixel level and bit level but can also be based on the deoxyribonucleic acid (DNA) level [39,40].

Blocks of pixels are higher-level data while DNA-level data and bit-level data are lower-level data. For the same processing power, an encryption employing lower-level data usually achieves better performance of encryption. The reason for this is that the lower the data level in this case, the more processed units will be involved in encryption [41]. Due to the vast parallelism, huge storage space, and ultra-low power consumption of DNA computing [42], some researchers have designed highly efficient and secure image encryption schemes using the technology of combining DNA operation and chaos [7,43,44]. Zhang and Han used an image hashing algorithm to generate the initial value and control parameter of a six-dimensional chaotic system and mainly combined it with DNA dynamic encoding and arithmetic operations for color image encryption [45]. Malik et al. presented a method with high plaintext sensitivity for color image encryption, which included a chaotic dynamical system and DNA computing, and combining permutation at the pixel level with diffusion at the DNA level [46]. Ref. [47] chose four-dimensional memristive chaos to generate chaotic matrices using the plain image, the salt key, and the control parameters and next executed dynamic DNA encoding via them; later, they applied dynamic confusion and diffusion to the encoded DNA matrices. Ref. [48] constructed an approach based on a five-dimensional chaotic system, pixel-level dynamic filtering, DNA computing, and operations on 3D Latin cubes and focused on transforming DNA-level images into several 3D DNA-level cubes to be computed using Latin cube. Zhou et al. proposed a dynamic DNA image encryption algorithm based on the secure hash algorithm-512 (SHA-512), which employed two chaotic systems, dynamic DNA encoding, DNA sequencing operations, and conditional shifting, and had two rounds of permutation with diffusion [49]. Chai et al. introduced a color image cryptosystem based on an improved genetic algorithm and matrix semi-tensor product, and they employed its adaptive block-based image preprocessing technology to deal with the red, green, and blue components of the color plain image; after DNA encoding, the resulting sequences can be shuffled via a double crossover operation of inter–intra components and diffused using a DNA complementary cycle mutation strategy [50]. Ref. [51] combined the quantum DNA codex with quantum Hilbert scrambling to present an enhanced quantum image encryption technique that offers high security with robustness and withstands statistical analysis and differential attacks. All of these schemes perform well for the high-security encryption of images.

In general, most DNA-level image encryption algorithms rely on the same DNA structure consisting of four bases. Traditional DNA encoding involves mapping ‘00’, ‘01’, ‘10’, and ‘11’ to the four DNA bases ‘A’, ‘T’, ‘C’, and ‘G’. These bases can be encoded in compliance with the complementary pairing principles. Nevertheless, researchers have actually succeeded in synthesizing DNA sequences using a stable double-helix structure for storing and transcribing genetic information in an eight-base system a few years ago, and the new set of artificial bases includes ‘S’, ‘B’, ‘P’, and ‘Z’ [52]. Notably, the encoding of eight-base DNA necessitates three bits and is far more varied in encoding rules that conform to the principles of complementary pairing of ‘A’ and ‘T’, ‘C’ and ‘G’, ‘S’ and ‘B’, and ‘P’ and ‘Z’ compared with the traditional four-base DNA. Thus, the utilization of eight-base DNA encoding and computation can be well suited for color image encryption.

Using the eight-base DNA structure, we propose a novel color image encryption scheme based on a four-dimensional hyperchaotic system, dynamic encoding and algebraic operations of eight-base DNA, and permutation and diffusion on eight-base DNA level data, termed as EDPD. Specifically, EDPD involves four main stages: (1) The secure hash algorithm-512 (SHA-512) combined with an input plaintext color image is applied to generate the initial values of the hyperchaotic system. Each color image is assigned a unique hyperchaotic sequence, derived from the four-dimensional hyperchaotic system using its specific initial values and control parameters. This sequence serves as the foundation for subsequent encryption. (2) The input image is transformed into a one-dimensional DNA sequence according to the eight-base DNA encoding rules determined using a subset of the chaotic sequence; then, the DNA sequence is permuted via the sorting indices of another chaotic sequence subset. After that, DNA-level diffusion is conducted, and then, the DNA sequence is permuted again via a different sorted chaotic subsequence. (3) The one-dimensional sequence is then converted into one or several DNA cubes. For each DNA cube, EDPD employs circular shifts of rows and columns as permutation and utilizes eight-base DNA-level algebraic operations as diffusion on three planes in each of the three axis directions per round, while each plane uses different eight-base DNA algebraic operation rules according to other hyperchaotic subsequences. (4) All the DNA cubes are integrated back into a singular one-dimensional sequence. Subsequently, the integrated sequence is decoded to a pixel-level color image via the rules represented by the hyperchaotic subsequence used at the time of encoding. It is important to note that, due to preceding permutations, the rules actually used for decoding each base at this time may be different from those used for encoding it before.

The main contributions of the proposed EDPD are threefold: (1) A newly proposed three-dimensional hyperchaotic system has been extended into four-dimensional version, which has better hyperchaotic attributes. All the subsequent operations of image encryption rely on the hyperchaotic sequence generated using this hyperchaotic system. (2) Eight-base DNA is introduced to color image encryption. Distinct encoding rules, decoding rules, and algebraic operations with eight-base DNA are designed for the permutation and diffusion of color images. Notably, the capability to encode three bits for each DNA base renders it exceptionally suitable for color image encryption involving three channels. (3) Extensive experiments demonstrate that the proposed EDPD is effective for color image encryption. The key innovation of this work is the application of the eight-base DNA to image encryption for the first time.

The structure of the remaining sections of this paper is as follows. In Section 2, a brief description of the original three-dimensional hyperchaotic system and the traditional DNA encoding with algebraic operations is summarized. In Section 3, a novel color image encryption algorithm based on the modified four-dimensional hyperchaotic system with the eight-base DNA-level multiple permutation and diffusion on different dimensions is presented in detail. In Section 4, extensive experiments are conducted on some common testing images to validate the effectiveness of our proposed EDPD, and the experimental results are reported and analyzed. Finally, in Section 5, the paper is concluded.

## 2. Related Work

### 2.1. Original Three-Dimensional Hyperchaotic System

Chaos has been extensively used in many research fields since Lorenz first found the chaotic attractor in 1963 [53]. In 2022, Wei et al. presented an improved three-dimensional chaotic system by integrating the logistic map and the ICMIC map through a closed-loop coupling mechanism for image encryption, which is formulated as Equation (1) [25].

(1) $$\left\{\begin{array}{c} x\left(i+1\right)=\cos\left[c\left(\frac{1}{ay(i)(1-y(i))}+\frac{1}{az(i)(1-z(i))}\right)\right]\sin\left[\frac{b}{x(i)}\right]\\ y\left(i+1\right)=\cos\left[c\left(\frac{1}{ax(i)(1-x(i))}+\frac{1}{az(i)(1-z(i))}\right)\right]\sin\left[\frac{b}{y(i)}\right]\\ z\left(i+1\right)=\cos\left[c\left(\frac{1}{ax(i)(1-x(i))}+\frac{1}{ay(i)(1-y(i))}\right)\right]\sin\left[\frac{b}{z(i)}\right] \end{array}\right.$$

In this system, *x*, *y*, and *z* are state variables and *a*, *b*, and *c* are constant parameters.

The fundamental characteristic of chaos is its extreme sensitivity to initial values, resulting in trajectories originating from even slightly distinct initial values diverging exponentially over time. A chaotic system has a Lyapunov exponent (LE) for each dimension. The LEs are used to measure the separation rate of infinitesimally close trajectories, which also reflect the expanding or contracting nature of the system between adjacent orbits in phase space [54]. A positive LE signifies a system that is both chaotic and expanding. When a system has two or more positive LEs, it can be regarded as a hyperchaotic system. The LEs of System (1) calculated with control parameters (*a, b, c*) = (0.1, π, π) and initial values (*x${}_{0}$, y${}_{0}$, z${}_{0}$*) = (0.3, 0.4, 0.5) on 50,000 iterations via the Jacobi matrix and QR method can obtain three results: $LE_{1}$ = 6.97688, $LE_{2}$ = 5.43797, and $LE_{3}$ = 3.88227 [55]. Therefore, System (1) is hyperchaotic. In addition, several chaotic systems proposed in recent years are also hyperchaotic [4,33,48].

### 2.2. Traditional DNA Computing

The traditional DNA molecule contains four distinct nucleic acid bases: adenine (‘A’), guanine (‘G’), cytosine (‘C’), and thymine (‘T’), where ‘A’ and ‘T’ are complementary and ‘G’ and ‘C’ are complementary. Since 0 and 1 in binary numbers are also complementary as DNA base pairing and each pixel value of the image can be represented by exactly one eight-bit binary number, images can be transformed from the pixel level to the bit level and then to the DNA level, where each pair of bits is converted to one base. Moreover, there are a total of 24 types of permutation via the utilization of the four bases ‘A’, ‘T’, ‘C’, and ‘G’ to encode ‘00’, ‘01’, ‘10’, and ‘11’. However, due to the complementary principles of both DNA bases and binary numbers, there are only eight types of DNA encoding rules that satisfy the Watson-Crick structure [56], as listed in Table 1.

According to these rules, DNA-level algebraic operations are able to resemble the operations in the binary number system, such as addition (⊕), subtraction (⊖), and XOR (⊗). These algebraic operations are usually applied to image encryption tasks as DNA-level diffusion. Moreover, due to multiple encoding rules, DNA algebraic operations differ from binary operations and can produce various results via different rules. In essence, DNA algebraic operations yield consistent results when executed in accordance with predetermined encoding rules. For example, the results of DNA addition, subtraction, and XOR operations based on encoding Rule 2 are listed in Table 2.

## 3. The Proposed Color Image Encryption Approach

### 3.1. Modified Four-Dimensional Hyperchaotic System

In general, a combined or more complex chaotic system has higher security of encryption than low-dimensional chaos. Therefore, we improve System (1) into a new four-dimensional one, formulated as Equation (2).

(2) $$\left\{\begin{array}{c} x\left(i+1\right)=\cos\left[c\left(\frac{1}{ay(i)(1-y(i))}+\frac{1}{az(i)(1-z(i))}+\frac{1}{aw(i)(1-w(i))}\right)\right]\sin\left[\frac{b}{x(i)}\right]\\ y\left(i+1\right)=\cos\left[c\left(\frac{1}{ax(i)(1-x(i))}+\frac{1}{az(i)(1-z(i))}+\frac{1}{aw(i)(1-w(i))}\right)\right]\sin\left[\frac{b}{y(i)}\right]\\ z\left(i+1\right)=\cos\left[c\left(\frac{1}{ax(i)(1-x(i))}+\frac{1}{ay(i)(1-y(i))}+\frac{1}{aw(i)(1-w(i))}\right)\right]\sin\left[\frac{b}{z(i)}\right]\\ w\left(i+1\right)=\cos\left[c\left(\frac{1}{ax(i)(1-x(i))}+\frac{1}{ay(i)(1-y(i))}+\frac{1}{az(i)(1-z(i))}\right)\right]\sin\left[\frac{b}{w(i)}\right] \end{array}\right.$$

**Figure 1:**
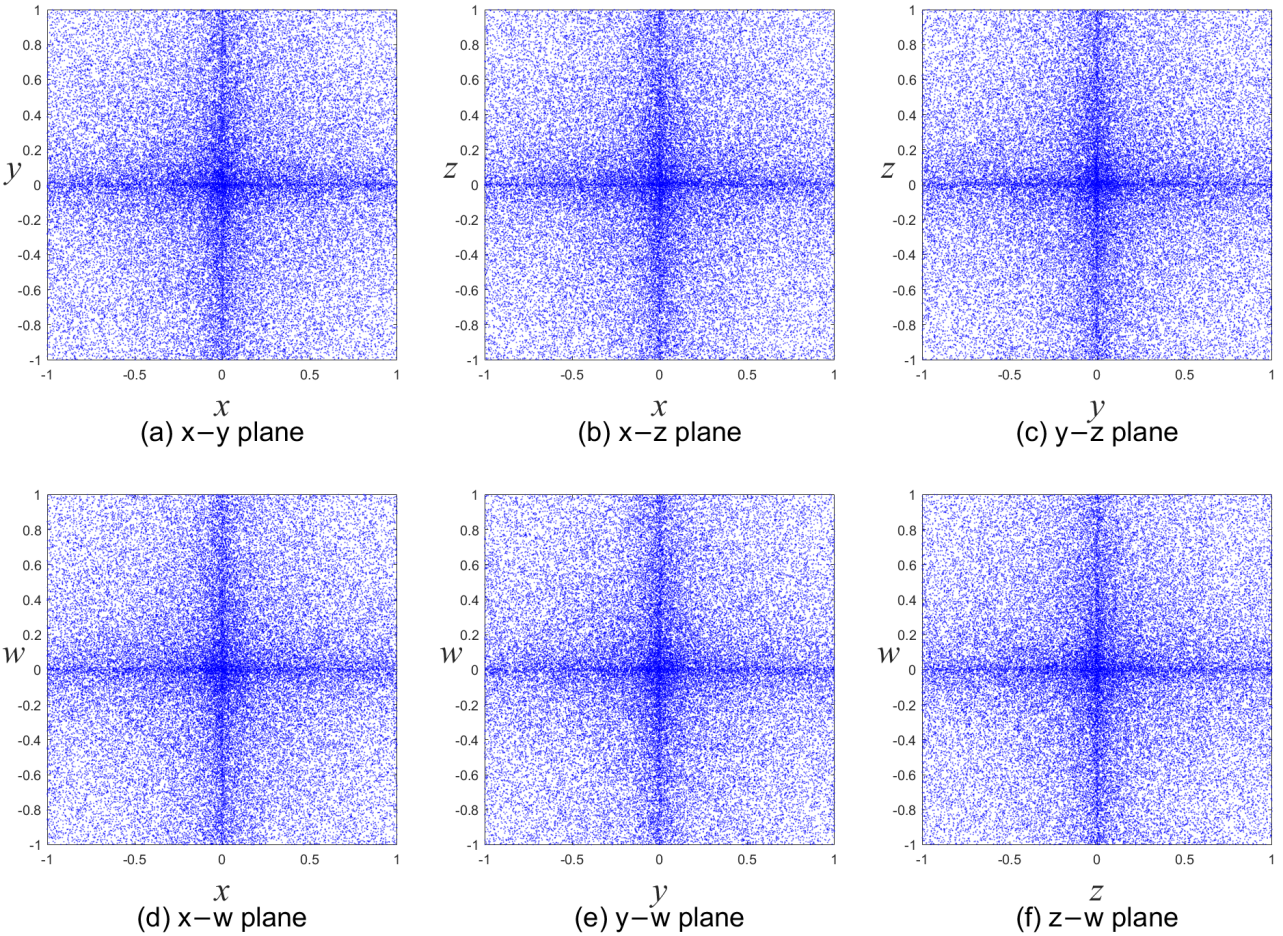
Attractors of the proposed four-dimensional hyperchaotic system with the parameters (*a, b, c*) = (0.1, 3, 2π) and initial values (*x${}_{0}$, y${}_{0}$, z${}_{0}$, w${}_{0}$*) = (0.3, 0.4, 0.5, 0.6) on 50,000 iterations.

In this system, the parameters *a*, *b*, and *c* retain their values from System (1), while the state variables are *x*, *y*, *z*, and *w*. We set the control parameters (*a, b, c*) = (0.1, 3, 2π) and initial values (*x${}_{0}$, y${}_{0}$, z${}_{0}$, w${}_{0}$*) = (0.3, 0.4, 0.5, 0.6) on 50,000 iterations for the proposed four-dimensional chaotic system. The chaotic attractors of System (2) are shown in Figure 1. The corresponding LEs are $LE_{1}$ = 8.34298, $LE_{2}$ = 6.62369, $LE_{3}$ = 5.44832, and $LE_{4}$ = 4.35038. Since the modified system has four positive LEs, it exhibits hyperchaotic characteristics. Furthermore, the first three LEs are greater than those of System (1).

**Figure 2:**
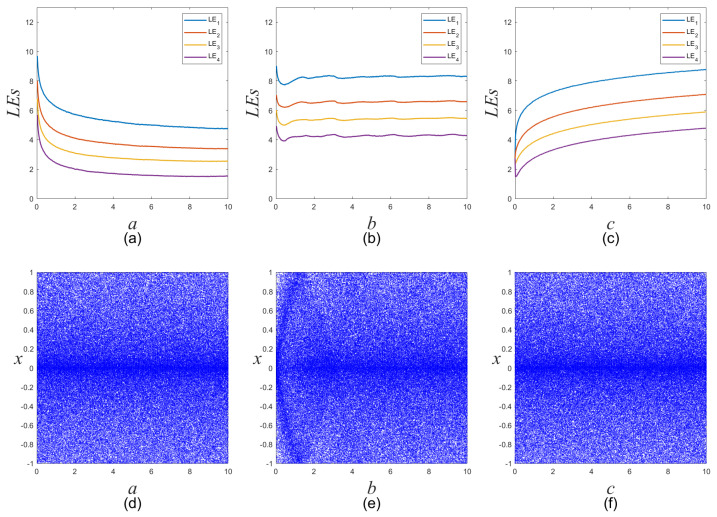
Spectra of Lyapunov exponents and bifurcation diagrams of the proposed four-dimensional hyperchaotic system with initial values (*x${}_{0}$, y${}_{0}$, z${}_{0}$, w${}_{0}$*) = (0.3, 0.4, 0.5, 0.6). (**a**,**d**) Spectra of Lyapunov exponents and bifurcation diagram with parameter *a* when *b* = 3, *c* = 2π. (**b**,**e**) Spectra of Lyapunov exponents and bifurcation diagram with parameter *b* when *a* = 0.1, *c* = 2π. (**c**,**f**) Spectra of Lyapunov exponents and bifurcation diagram with parameter *c* when *a* = 0.1, *b* = 3.

In order to investigate the influence of each control parameter variation on the performance of the System (2), the spectra of LEs and bifurcation diagrams are plotted in Figure 2a–c and Figure 2d–f, respectively. As indicated by these figures, the four LEs consistently remain positive under these conditions. Specially, as the parameter *a* becomes smaller or the parameter *c* becomes larger, the four LEs and the performance of the hyperchaotic system keep increasing. And, when the parameter *b* > 1, the four LEs maintain stability. In addition, no bifurcation is observed within these figures, and the distribution of the state variables consistently reflects chaotic properties. In summary, owing to its sensitivity to initial values and complex dynamical behaviors, the modified four-dimensional hyperchaotic system is highly suitable for color image encryption.

### 3.2. The Presented Eight-Base DNA Computing

Hoshika et al. synthesized the novel eight-base DNA sequences in 2019, which can store and transcribe genetic information with stable double-helix structures like conventional DNA sequences [52]. In the eight-base DNA structure, in addition to the traditional four bases ‘A’, ‘T’, ‘C’, and ‘G’, there are the other four artificial bases ‘S’, ‘B’, ‘P’, and ‘Z’. The principles of complementary pairing in the eight-base DNA are ‘A’ with ‘T’, ‘C’ with ‘G’, ‘S’ with ‘B’, and ‘P’ with ‘Z’. Consequently, for eight-base DNA encoding, each three bits are converted to a single base, which are ‘000’, ‘001’, ‘010’, ‘011’, ‘100’, ‘101’, ‘110’, and ‘111’. Although there are a total of 40,320 possible permutations of the eight bases, only 384 ones conform to the complementary pairing principles of eight-base DNA. Obviously, the total number of encoding rules for eight-base DNA significantly surpasses that of conventional DNA. Ten of these encoding rules are listed in Table 3.

Based on the traditional DNA algebraic operations, the eight-base DNA-level addition (⊕), subtraction (⊖), and XOR (⊗) are able to resemble the corresponding operations in the three-bit binary number. However, owing to the far more varied eight-base DNA encoding rules, the algebraic operations between two bases become more complex. For example, the results of these algebraic operations based on eight-base DNA encoding Rule 1 are listed in Table 4, Table 5 and Table 6. Since the more bases and much richer encoding rules make the algebraic operations between bases more varied and complex, using an eight-base DNA-level encryption scheme can bring more benefits for image security.

### 3.3. Hyperchaotic Sequence Generation

Encryption methods that use the same key for multiple different images are vulnerable to known-plaintext and chosen-plaintext attacks. To enhance the ability of resisting these risks, the proposed EDPD introduces the information of input images into their keys to make each image have a unique key. In this way, it is difficult for attackers to break the keys even if they are analyzed by using some existing or optionally generated plaintext-ciphertext pairs because the generation of the each key introduces information of a plain image itself thus disturbing the regularity. Specifically, the SHA-512 algorithm is used to calculate the hash value from a plaintext image. This hash value is then divided into four parts, each of which contributes to generating the initial values of the hyperchaotic system. This approach ensures that every color image obtains its specific initial values. Subsequently, we can use the control parameters and four initial values to generate the hyperchaotic sequence via System (2). The whole procedure is described as follows:

Step 1: Convert the plain color image into a one-dimensional sequence, and calculate its SHA-512 hash value, denoted as *K*.
Step 2: Divide the hash value *K* into 64 blocks, where each block contains an eight-bit integer.
Step 3: Express the 64 blocks of *K* as *K* = {$k_{1}$, $k_{2}$, ⋯, $k_{64}$}. Due to a total of four initial values of System (2), there are four intermediate parameters $t_{1}$, $t_{2}$, $t_{3}$, and $t_{4}$ calculated using Equation (3).
  (3) $$\left\{\begin{array}{c} t_{1}=b_{1}+\frac{1}{256}\left(k_{1}⊗k_{2}⊗\cdots ⊗k_{16}\right)\\ t_{2}=b_{2}+\frac{1}{256}\left(k_{17}⊗k_{18}⊗\cdots ⊗k_{32}\right)\\ t_{3}=b_{3}+\frac{1}{256}\left(k_{33}⊗k_{34}⊗\cdots ⊗k_{48}\right)\\ t_{4}=mean\left(b_{1}+b_{2}+b_{3}\right)+\frac{1}{256}\left(k_{49}⊗k_{50}⊗\cdots ⊗k_{64}\right) \end{array}\right.$$
  where $b_{1}$, $b_{2}$, and $b_{3}$ are three user-defined real-number parameters, and *mean* and ⊗ represent the average and bitwise XOR operation, respectively.
Step 4: Employ the intermediate parameters $t_{1}$, $t_{2}$, $t_{3}$, and $t_{4}$ to obtain the initial values $x_{0}$, $y_{0}$, $z_{0}$, and $w_{0}$ of the four-dimensional hyperchaotic system via Equation (4).
  (4) $$\left\{\begin{array}{c} x_{0}=\frac{mod(\left(t_{2}+t_{3}+t_{4}\right)\;\times \;10^{8},\;256)}{255}\\ y_{0}=\frac{mod(\left(t_{1}+t_{3}+t_{4}\right)\;\times \;10^{8},\;256)}{255}\\ z_{0}=\frac{mod(\left(t_{1}+t_{2}+t_{4}\right)\;\times \;10^{8},\;256)}{255}\\ w_{0}=\frac{mod(\left(t_{1}+t_{2}+t_{3}\right)\;\times \;10^{8},\;256)}{255} \end{array}\right.$$
  where *mod* represents the module operation. In case one or more of the four initial values $x_{0}$, $y_{0}$, $z_{0}$, and $w_{0}$ are 0 or 1, these values should be replaced with the average of the other initial values. If they are all 0 or 1, make them equal to $\frac{1}{\pi }\arctan\left(a\right)+\frac{1}{2}$, $\frac{1}{\pi }\arctan\left(b\right)+\frac{1}{2}$, $\frac{1}{\pi }\arctan\left(c\right)+\frac{1}{2}$, and $\frac{1}{\pi }\arctan\left(a+b+c\right)+\frac{1}{2}$ in turn, where *a*, *b*, and *c* are the System (2). Thus, all initial values fall within the range of (0, 1), enabling the System (2) to iterate the computation properly.
Step 5: Input the control parameters *a*, *b*, and *c* with the initial values $x_{0}$, $y_{0}$, $z_{0}$, and $w_{0}$ into System (2) to generate a hyperchaotic matrix, and then, transform it into a hyperchaotic sequence as *S* for the subsequent encryption of the plain color image. In addition, the length of the sequence is the number of elements within it and labeled as *L* = *h* × *w* × *d* × 8, where *h*, *w*, and *d* denote the height, width, and number of channels of the input color image, respectively.
Step 6: According to System (2) and Figure 1, the hyperchaotic sequence *S* should take values within the range of [−1, 0)∪(0, 1). To prepare for the subsequent color image encryption, extract two subsequences as $S_{1}^{p}$ and $S_{2}^{p}$ of length *L*/3 from the hyperchaotic sequence *S*, and then, adjust the values of the hyperchaotic sequence *S* to integers within 1 to 384 using Equation (5).
  (5) $$S=\lfloor (|S\times 10^{8}|-\lfloor |S\times 10^{8}|\rfloor )\times 384\rfloor +1$$
  where |·| and ⌊·⌋ denote the operations of absolute and rounding down.

### 3.4. The Permutation and Diffusion at One-Dimensional Sequence Level

Prior to the permutation and diffusion, the plain color image is encoded into eight-base DNA level by using a hyperchaotic subsequence $S_{ed}$ to determine the eight-base DNA encoding rules dynamically for each three bits. The length of the resulting DNA sequence *D* is *L*/3. The subsequent permutation and diffusion operations performed on the one-dimensional sequence *D* comprise the following steps:

Step 1: Arrange the hyperchaotic subsequence $S_{1}^{p}$ in ascending order, thereby obtaining the indices of the sorted elements in the original sequence. Subsequently, use these new indices to scramble the positions of bases in the DNA sequence *D* as the first permutation.
Step 2: Extract a subsequence $S_{d}$ with length *L*/3 from the hyperchaotic sequence *S*, and use the elements in $S_{d}$ to determine the eight-base DNA algebraic operation rules on the DNA sequence *D*. The simple diffusion process at one-dimensional sequence level is described using Equation (6).
  (6) $$D_{i}=\left\{\begin{array}{cc} D_{i}⊕D_{L_{D}}, & i=1\\ D_{i}⊕D_{i-1}, & 1<i\leq L_{D} \end{array}\right.$$
  where $D_{i}$ represents a certain base in the DNA sequence *D*, $L_{D}$ denotes the length of the DNA sequence *D*, and ⊕ indicates eight-base DNA-level addition with different algebraic operation rules.
Step 3: Referring to Step 1, sort the hyperchaotic subsequence $S_{2}^{p}$ for scrambling the DNA sequence *D* again as the second permutation.

On the one-dimensional sequence, the first permutation performs a global position disruption of the eight-base DNA sequence *D*, and the eight-base DNA-level diffusion makes the algebraic operation of each base associated with its last base, while the second permutation extends the effects of diffusion to the whole eight-base DNA sequence *D*.

### 3.5. The Permutation at Three-Dimensional Cube Level

After the permutation and diffusion at the one-dimensional sequence level, the subsequent phase involves conducting eight-base DNA-level operations at the three-dimensional cube level. Thus, the eight-base DNA sequence *D* requires reshaping to one or several cubes. Specifically, given the length $L_{D}=L$/3 of the eight-base DNA sequence encoded from the input color image, if the result of $\sqrt[{3}]{L_{D}}$ is an integer, the prism length of the only cube is $E=\sqrt[{3}]{L_{D}}$. Otherwise, $\sqrt[{3}]{L_{D}/2^{N}}$ keeps running until its value becomes an integer, where *N* is an increasing positive integer, and the prism length of each of the $2^{N}$ cubes is $E=\sqrt[{3}]{L_{D}/2^{N}}$ in the end. For example, a color image with size 512 × 512 × 3 can be transformed into an eight-base DNA-level cube with size 128 × 128 × 128, while another color image with size 256 × 256 × 3 will become two eight-base DNA-level cubes both with size 64 × 64 × 64.

In this encryption session, the details of permutation and diffusion on each eight-base DNA-level cube are the same. For each cube *C*, we first execute the permutation, and the scheme is based on the hyperchaotic subsequences and circular shifts.

**Figure 3:**
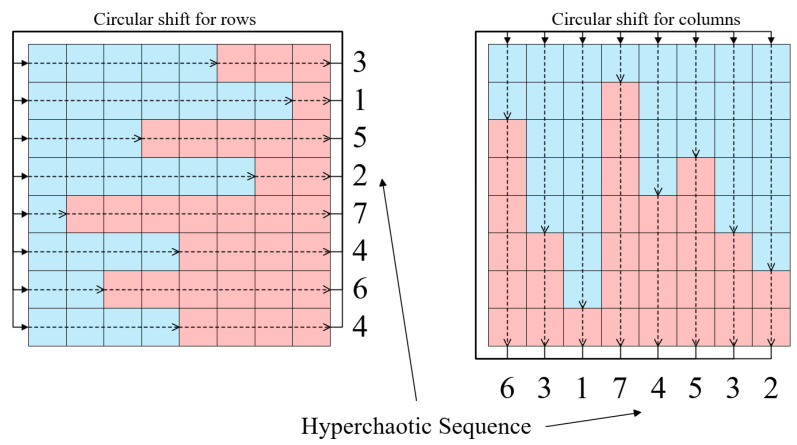
Schematic diagram of circular shifts of rows and columns in a plane. The numbers indicate the step size of the circular shift. The dashed lines and solid arrows show the shifting direction. The pixels in the blue boxes are shifted to the right or down, while those in the pink ones are circularly shifted to the left or top.

Circular shift is a very useful method of permutation that usually includes operations on rows and columns within a plane, as shown in Figure 3. More specifically, the number of steps in each row or column circular shift is generally determined using the values of hyperchaotic sequence. Then, for each row, the corresponding number of pixels on the right side are circularly moved to the left side in turn according to the number of steps, while the pixels originally on the left side are moved to the right side in turn simultaneously. Similarly, for each column, some pixels at the bottom are circularly moved to the top in order, while those at the top are pushed to the bottom. The permutation on *C* is described in Algorithm 1 in detail.

**Algorithm 1** The permutation on a single eight-base DNA-level cube**Input:** an eight-base DNA-level cube *C*, the prism length *E* of the cube, and three hyperchaotic subsequences $H_{j}^{p}$ (*j* = 1, 2, 3)**Output:** the eight-base DNA-level cube *C* after the permutation1: **function** CubePermutation($C,E,H_{j}^{p}$)2:     **for** *i* = 1 : *E* **do**3:         **for** *j* = 1 : 3 **do**4:           $P_{i,1}\leftarrow C\left(i,:,:\right)$;5:            $P_{i,2}\leftarrow C\left(:,i,:\right)$;6:            $P_{i,3}\leftarrow C\left(:,:,i\right)$;7:            **for** *t* = 1 : *E* **do**8:                $Shifts\leftarrow H_{j}^{p}\left(2\times \left(i-1\right)\times E+t\right)$;9:                Circular shift $P_{i,j}\left(t,:\right)$ to right by *Shifts*;10:            **end for**11:            **for** *t* = 1 : *E* **do**12:                $Shifts\leftarrow H_{j}^{p}\left(2\times \left(i-1\right)\times E+E+t\right)$;13:                Circular shift $P_{i,j}\left(:,t\right)$ to bottom by *Shifts*;14:            **end for**15:            $C\left(i,:,:\right)\leftarrow P_{i,1}$;16:            $C\left(:,i,:\right)\leftarrow P_{i,2}$;17:            $C\left(:,:,i\right)\leftarrow P_{i,3}$;18:         **end for**19:     **end for**20:     **return** *C*;21: **end function**

Step 1: For each eight-base DNA-level cube *C*, extract a subsequence with a length of 6 × $E^{2}$ from the hyperchaotic sequence *S*, and then split this subsequence into three subsequences with a length of 2 × $E^{2}$ each, which are designated as $H_{1}^{p}$, $H_{2}^{p}$, and $H_{3}^{p}$, respectively. Finally, the values of these subsequences are scaled to range between 1 and E−1.
Step 2: Start the iteration with a total number of rounds *E* that represents the quantity of the planes of each axis direction in a cube, and perform the permutation for the current planes of the three axis directions in turn per round. For each current plane, execute circular shifts on all rows and all columns of DNA bases in turn by using the elements in the hyperchaotic subsequence $H_{1}^{p}$, $H_{2}^{p}$, or $H_{3}^{p}$ in order.
Step 3: Reiterate Step 2 until the end of the iteration, which completes the permutation on a single cube *C*.

If there are multiple cubes, the permutation details of remaining cubes follow the same process as described above.

### 3.6. The Diffusion at Three-Dimensional Cube Level

After the permutation of the current eight-base DNA-level cube *C*, the subsequent step involves diffusion. In this way, besides the three hyperchaotic subsequences used to determine the algebraic operation rules between any two bases of eight-base DNA, there is also an auxiliary eight-base DNA-level cube with the same size as *C* that is encoded and transformed from another hyperchaotic subsequence. The specific diffusion process of *C* is explained in Algorithm 2 and is described as follows.

**Algorithm 2** The diffusion on a single eight-base DNA-level cube**Input:** an eight-base DNA-level cube *C* after the permutation, an auxiliary hyperchaotic eight-base DNA-level cube *G*, the same prism length *E* of the cube *C* and *G*, and three hyperchaotic subsequences $H_{j}^{d}$ (*j* = 1, 2, 3)**Output:** the eight-base DNA-level cube *C* after the diffusion1: **function**
CubeDiffusion($C,G,E,H_{j}^{d}$)2:     C(1,:,:)=(C(1,:,:)⊕G(E,:,:))⊗(C(E,:,:)⊕G(1,:,:)) with Rule $H_{1}^{d}\left(1\right)$;3:     C(:,1,:)=(C(:,1,:)⊕G(:,E,:))⊗(C(:,E,:)⊕G(:,1,:)) with Rule $H_{2}^{d}\left(1\right)$;4:     C(:,:,1)=(C(:,:,1)⊕G(:,:,E))⊗(C(:,:,E)⊕G(:,:,1)) with Rule $H_{3}^{d}\left(1\right)$;5:     **for** *i* = 2 : *E* **do**6:            C(i,:,:)=(C(i,:,:)⊕G(i−1,:,:))⊗(C(i−1,:,:)⊕G(i,:,:)) with Rule $H_{1}^{d}\left(i\right)$;7:            C(:,i,:)=(C(:,i,:)⊕G(:,i−1,:))⊗(C(:,i−1,:)⊕G(:,i,:)) with Rule $H_{2}^{d}\left(i\right)$;8:            C(:,:,i)=(C(:,:,i)⊕G(:,:,i−1))⊗(C(:,:,i−1)⊕G(:,:,i)) with Rule $H_{3}^{d}\left(i\right)$;9:     **end for**10:   **return** *C*;11: **end function**

Step 1: For each eight-base DNA-level cube *C*, extract a subsequence with a length of 3 × *E* from the hyperchaotic sequence *S*, and then, split this subsequence into three subsequences with a length of *E*, which are denoted as $H_{1}^{d}$, $H_{2}^{d}$, and $H_{3}^{d}$, respectively. Moreover, obtain another subsequence of length *L*/3 from the hyperchaotic sequence *S*, and scale the values of this subsequence to a range between 0 and 7 via the module operation. Then, transform these values of the subsequence into three-bit binary numbers, and then, dynamically encode them into eight-base DNA sequence by the hyperchaotic subsequence $S_{ed}$. Finally, reshape them into one or more three-dimensional cubes *G* with the same size as the eight-base DNA-level cube *C*.
Step 2: Start the iteration with a total number of rounds *E* that represent the number of the planes of each axis direction in a cube, and perform the diffusion for the current planes of the three axis directions in turn per round. For each plane of the eight-base DNA-level cube *C*, eight-base DNA algebraic operations are performed on it with *G* at corresponding positions of the planes. Specifically, the corresponding DNA base positions between each *C* and *G*, along with the details of DNA algebraic operations are depicted in Equation (7).
  (7) $$C_{i,j}=\left\{\begin{array}{cc} \left(C_{i,j}⊕G_{E,j}\right)⊗\left(C_{E,j}⊕G_{i,j}\right), & i=1\\ \left(C_{i,j}⊕G_{i-1,j}\right)⊗\left(C_{i-1,j}⊕G_{i,j}\right), & 1<i\leq E \end{array}\right.$$
  where *j* denotes the direction of the current axis of the cube and takes values of 1, 2, or 3. $C_{i,j}$ and $G_{i,j}$ represent the planes corresponding to cubes *C* and *G* at position *i* in the *j*-axis direction. And, the algebraic operation rules of the eight-base DNA in the *E* planes in each axis direction are determined using the elements in $H_{1}^{d}$, $H_{2}^{d}$, and $H_{3}^{d}$, respectively.
Step 3: Continue executing Step 2 until the end of the iteration to complete the diffusion on a single cube *C*.

If there are multiple cubes, the diffusion algorithm for the remaining cubes is the same as the above process. After completing all the permutation and diffusion rounds on the eight-base DNA-level cubes, they are transformed into a one-dimensional sequence and decoded using the hyperchaotic subsequence $S_{ed}$. Since the positions of the DNA bases have been changed using the permutation, they actually mostly use different encoding and decoding rules. Finally, the bit sequence obtained via eight-base DNA decoding is converted back to the pixel level and the pixel sequence is reshaped into a cipher color image.

### 3.7. Framework of the Proposed EDPD

**Figure 4:**
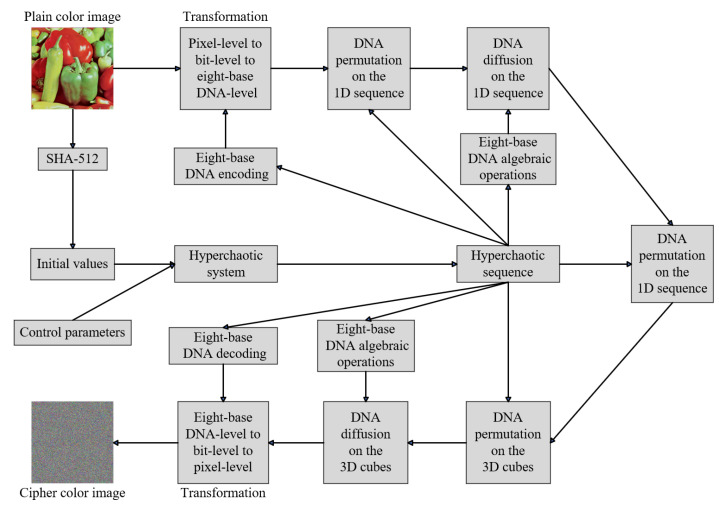
The framework of the proposed EDPD.

The permutation and diffusion of the proposed color image encryption scheme EDPD are conducted on the eight-base DNA level. We use a total of two stages of both the permutation and diffusion sessions, and they are designed and applied at the one-dimensional sequence and three-dimensional cube levels, respectively. Moreover, the encoding rules, decoding rules, and algebraic operation rules are dynamically determined using the hyperchaotic subsequences. In summary, the flowchart of the proposed EDPD is illustrated in Figure 4, wherein we define the execution of all steps from the plaintext color image to the cipher color image as a single round of encryption, symbolized as *R*.

Step 1: Combine the SHA-512 algorithm with an input plaintext color image to generate the four initial values of the hyperchaotic system (2).
Step 2: Input the three control parameters and four initial values into the hyperchaotic system in (2) to generate a hyperchaotic sequence *S*. Preserve two hyperchaotic subsequences, $S_{1}^{p}$ and $S_{2}^{p}$, for the permutation at the subsequent one-dimensional sequence level, and adjust the values of the whole hyperchaotic sequence *S* to fall within 1 to 384.
Step 3: Transform the plain color image from the pixel level to the bit level, and use a hyperchaotic subsequence $S_{ed}$ for dynamic eight-base DNA encoding of the bit-level image, where every three bits are converted into one base according to the corresponding encoding rule. Thus, the bit-level image is transformed into a one-dimensional eight-base DNA sequence *D*.
Step 4: Sort the hyperchaotic subsequence $S_{1}^{p}$ in ascending order, and then, use the indices to scramble the positions of bases in the one-dimensional eight-base DNA sequence *D*.
Step 5: Extract a hyperchaotic subsequence $S_{d}$ from *S* to dynamically determine the eight-base DNA algebraic operation rules between two eight-base DNA bases. Then, apply it to the diffusion at the one-dimensional sequence level for each iteration round using Equation (6).
Step 6: Sort the hyperchaotic subsequence $S_{2}^{p}$ in ascending order, and then, use the indices to perform global permutation.
Step 7: Reshape the eight-base DNA sequence *D* into one or more cubes with a prism length *E*, and obtain three hyperchaotic subsequences $H_{1}^{p}$, $H_{2}^{p}$, and $H_{3}^{p}$ from *S*, whose values are all scaled into the range 1 to E−1. For each eight-base DNA-level cube *C*, employ the Algorithm 1 with these hyperchaotic subsequences for the permutation.
Step 8: Transform and reshape a hyperchaotic subsequence into one or more eight-base DNA-level cubes *G* by using the hyperchaotic subsequence $S_{ed}$ for the dynamic eight-base DNA encoding, and split another hyperchaotic subsequence into three subsets $H_{1}^{d}$, $H_{2}^{d}$ and $H_{3}^{d}$. Input these hyperchaotic subsequences, as well as each cube *C* and *G* into Algorithm 2 to execute the diffusion.
Step 9: Transform the one or more three-dimensional eight-base DNA-level cubes back to a one-dimensional sequence, and decode it into a pixel-level color cipher image using the hyperchaotic subsequence $S_{ed}$.

The proposed EDPD consists of five parts: hyperchaotic sequence generation (Steps 1–2), transformation from the pixel level to the eight-base DNA level (Step 3), two permutation steps and a diffusion step between them at the one-dimensional sequence level (Steps 4–6), permutation and diffusion alternation at the three-dimensional cube level (Steps 7–8), and transformation from the eight-base DNA level back to the pixel level to obtain the final color cipher image (Step 9). These parts are visually depicted in Figure 4. Due to all encryption stages being at the eight-base DNA level, the permutation operations that modify the positions of bases of eight-base DNA change the corresponding values of the pixels naturally and result in the diffusion of pixels.

By using SHA-512, the information carried by the input plaintext color image influences the generation of the initial values of the hyperchaotic system, which can in turn generate a unique hyperchaotic sequence to enhance resistance against known-plaintext and chosen-plaintext attacks. Moreover, the eight-base DNA encoding has 384 rules, a significant increase compared with the traditional DNA encoding’s eight rules. Applying these rules for dynamic encoding can significantly improve the performance and security of encryption. The subsequent permutation and diffusion at both the one-dimensional sequence level and three-dimensional cube level enhance resistance against differential attacks while bolstering the security and robustness of color image encryption.

The decryption process for the color cipher image is the inverse of the above steps. It needs to reverse the plain image with cipher image and the DNA encoding with decoding stages in Figure 4. Furthermore, all permutation and diffusion stages are executed in reverse order, and the traversal operations in them have to be reversed as well. In particular, during eight-base DNA algebraic operations in decryption, DNA additions employed in encryption are replaced with DNA subtractions, while DNA XOR operations remain unchanged.

## 4. Experimental Results

### 4.1. Experimental Settings

To evaluate the performance of the proposed EDPD, we conducted a series of experiments and compared it with other recent state-of-the-art color image encryption methods. Specifically, we list all the parameters used in these experiments in Table 7. The user-defined parameters $b_{1}$, $b_{2}$, and $b_{3}$ in Equation (3) were used to generate the initial values of the proposed four-dimensional hyperchaotic system. In the experiments, we set the values of *a*, *b*, and *c* to be exactly equal to $b_{1}$, $b_{2}$, and $b_{3}$, respectively. In addition, considering the length *L* = *h* × *w* × *d* × 8 of the final hyperchaotic sequence, we set the number of iterations for generating a hyperchaotic sequence to *n* = *h* × *w* × *d* × 2−1, where *h*, *w* and *d* represent the height, width, and number of channels of the plain color image, respectively. In particular, we set the total number of complete encryption operations once. Performing multiple iterations would require a longer hyperchaotic sequence, leading to a significant increase in encryption time.

Eight RGB color images were used as test images for our experiments, as detailed in Table 8. All images share a common size of 512 × 512 × 3 pixels. Notably, the first five images are widely used standard test images, while the last three are available from Ref. [51].

All experiments were conducted using MATLAB R2021b (Mathworks, Natick, MA, USA) on a PC with 64-bit Windows 11 OS (Microsoft, Redmond, WA, USA), an R7-5800H CPU at 3.20 GHz, and 16 GB RAM.

### 4.2. Security Key Analysis

Security keys play a pivotal role in image encryption, and an excellent image encryption scheme should have a large key space and high key sensitivity, which can resist brute-force attacks.

#### 4.2.1. Key Space

An effective image encryption approach should possess a key space of considerable size to resist brute-force attacks. If the size of a key space is larger than $2^{100}$, it can provide enough security against brute-force attacks carried out with modern computing capabilities. In the proposed EDPD, the four initial values $x_{0}$, $y_{0}$, $z_{0}$, and $w_{0}$ of the hyperchaotic system (2) can be employed as the security keys. If the precision of each security key with a floating point type is $10^{-15}$, the total key space is $10^{15\times 4}=10^{60}\approx 2^{199}$, which is much larger than $2^{100}$. Thus, the EDPD is excellent at resisting brute-force attacks. Moreover, by incorporating the hash value of the input plaintext color image, the iteration count of the hyperchaotic system, and even the number of encryption rounds as additional security keys, the key space can be easily further expanded.

#### 4.2.2. Sensitivity to Security Keys

**Figure 5:**
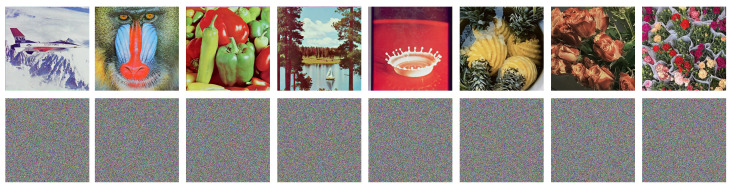
Decrypted images with security keys $g_{1}$ and $g_{2}$. The **first row** and the **second row** are with $g_{1}$ and $g_{2}$, respectively. From **left** to **right**: Airplane, Baboon, Peppers, Sailboat, Splash, Pineapple, Rose, and Plants.

An effective image encryption method must exhibit a high level of sensitivity to the security keys. In other words, when the security keys change extremely slightly, the resulting decrypted image will be completely different from the correct plain image. In the proposed EDPD, the security keys are associated with the input plaintext color images. For the key sensitivity test, we set up two groups of slightly different security keys for each testing image to decrypt the corresponding cipher image, which are $g_{1}=\left(x_{0},y_{0},z_{0},w_{0}\right)$ and $g_{2}=\left(x_{0}+10^{-15},y_{0},z_{0},w_{0}\right)$. The decrypted results obtained using $g_{1}$ and $g_{2}$, respectively, are shown in Figure 5. Obviously, even an exceedingly tiny variation of $10^{-15}$ between the two security key sets leads to a vastly distinct decryption outcome and make the cipher image impossible to be recovered correctly. Therefore, the proposed EDPD demonstrates a notable sensitivity to the security keys.

### 4.3. Statistical Analysis

Statistical analysis typically includes histogram analysis, information entropy analysis, and correlation analysis. An effective image encryption scheme should ensure that the cipher images have high information entropies, flat histograms, and low correlations. In this way, the ideal image encryption algorithm can effectively resist statistical attacks.

#### 4.3.1. Histogram Analysis

**Figure 6:**
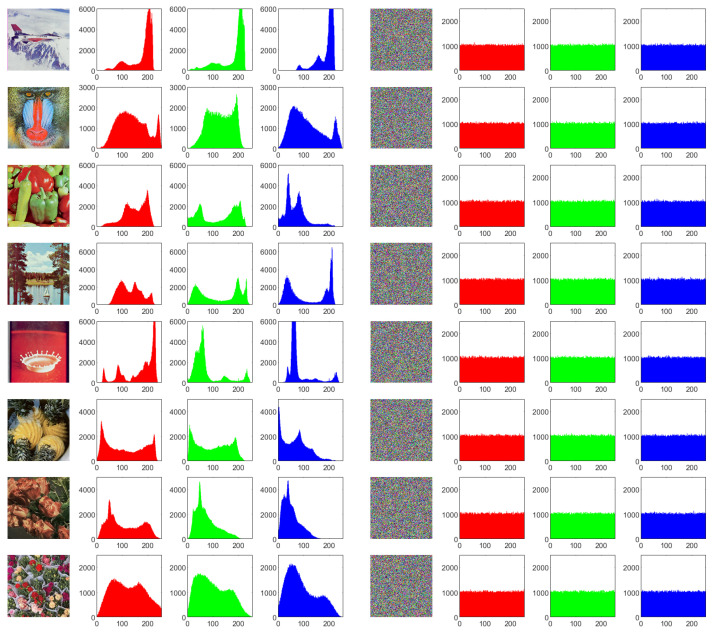
Images and histograms. The **first** to the **fourth columns** are, respectively, the plain images and their histograms on the three channels R, G, and B. The **fifth** to the **last columns** are, respectively, the corresponding cipher images and their histograms on the three channels R, G, and B.

The histogram of an image records the number of times each pixel appears, which reflects pixel-level frequency distribution. In general, natural images must have an uneven distribution of pixels; thus, their histograms are irregular. In image encryption tasks, the histograms of plain images commonly contain irregular shapes such as undulating peaks and valleys. A qualified image encryption approach should transform the original uneven distribution of pixels to flatten the histograms of cipher images as much as possible. The histograms of plain color images and their corresponding cipher images generated using EDPD are shown in Figure 6.

From the figure, it can be found that the histograms of plain color image channels are shown uneven and irregular pixel-level frequency distributions. However, the histograms of the three color channels of almost all the corresponding cipher images are very flat and similar. Although the histograms of the plain color images differ significantly, the corresponding cipher images share uniform and closely matching histograms, with pixel values occurring roughly 1000 times. According to the results of the histograms, it can be concluded that the cipher images produced using EDPD have quite uniform and flat pixel-level frequency distribution. Therefore, EDPD is highly effective at resisting histogram attacks.

#### 4.3.2. Information Entropy

Information entropy (IE) is a fundamental concept in information theory, which is proposed to solve the problem of quantitative measurement of information. In general, it is often used as an indicator to describe the uncertainty and randomness of a complex system, which can be also explained as the probability of occurrence of discrete random events. Given a single channel *C* of a color image with $2^{8}=256$ gray-scale levels, its IE can be calculated using Equation (8).

(8) $$IE\left(C\right)=-\sum_{i=0}^{255}p_{i}log_{2}p_{i}$$

where $p_{i}$ denotes the probability of gray-scale level *i* appearing in the whole channel *C*. When there is only one gray-scale level, the IE attains its minimal value of 0. If every gray-scale appears with equal probability, $\frac{1}{256}$, the IE obtains its maximal value of 8. In fact, the IE of each channel of a natural color image is usually less than 8. Therefore, an excellent color image encryption scheme should aim for an IE value as close to 8 as possible for each channel. In this way, all histograms of the cipher images can be very uniform and flat at the same time.

We list the IEs of the testing images and their corresponding cipher images using the proposed EDPD and the other compared color image encryption schemes in Table 9. It can be seen that the IEs of these plain color images are far below the maximum theoretical value 8, while the IEs of all the cipher images except for the method of Ref. [51] achieve a range from 7.9992 to 7.9994. Moreover, among the approaches, the proposed EDPD obtains the highest IEs in 13 out of 24 cases and has the most occurrences, followed by the scheme of Ref. [36], with 12 occurrences; that of Ref. [31], with 11; that of Ref. [10], with 10; that of Ref. [34], with 8 out of 15 cases; and that of Ref. [51], with 2 out of 9 cases. Therefore, the presented EDPD is advantageous over or comparable with other approaches in terms of IE, effectively resisting entropy attacks.

#### 4.3.3. Correlation Analysis

High correlations exist among neighboring pixels of natural images, which means these pixels are very similar or even identical. An excellent image encryption method should break such a situation to make the correlations in the cipher images significantly decrease, ideally to the point of near-zero correlation. To measure the correlations in the images, the correlation coefficient γ is defined as Equation (9).

(9) $$\begin{array}{c} E\left(x\right)=\frac{1}{M}\sum_{i=1}^{M}x_{i}\\ D\left(x\right)=\frac{1}{M}\sum_{i=1}^{M}{\left(x_{i}-E\left(x\right)\right)}^{2}\\ cov\left(x,y\right)=\frac{1}{M}\sum_{i=1}^{M}\left(x_{i}-E\left(x\right)\right)\left(y_{i}-E\left(y\right)\right)\\ \gamma =\frac{cov(x,y)}{\sqrt{D(x)D(y)}} \end{array}$$

In this equation, *x* and *y* are gray-scale levels of two adjacent pixels in a channel of a color image; *M* is the total number of pixel pairs; and E(x), D(x), and cov(x,y) denote the expectation of *x*, the standard deviation of *x*, and the covariance of *x* and *y*, respectively.

Calculating the correlations of an image or a certain color channel needs to involve all the current pixels. The correlations of the plain images and cipher images are presented in Table 10, where $\gamma_{h}$, $\gamma_{v}$, and $\gamma_{d}$ represent the correlation at the horizontal, vertical, and diagonal directions, respectively. Since correlation coefficients can be positive or negative, the lowest absolute values of the correlation coefficients are shown in bold for each case, which indicates the best results. From the table, it is clear that all the plain color images have high correlation coefficients close to 1 in each channel, showing that there are strong correlations in the plain color images. Conversely, all the corresponding cipher images obtain very low correlations, which indicates that these encryption schemes have the ability to break the strong correlations in the plain color images. The experimental results show that the absolute values of the correlation coefficients of the cipher images are mostly close to 0. Among all results in the table, our proposed EDPD achieves the lowest correlations in 21 out of 72 cases and has the highest occurrences, followed by the scheme of Ref. [31] with 20 cases; those of Refs. [10,36], both with 16 cases; that of Ref. [34], with 1 out of 45 cases; and that of Ref. [51], with 1 out of 27 cases. Thus, the comparison results validate that our proposed EDPD achieves superior performance in terms of correlation.

**Figure 7:**
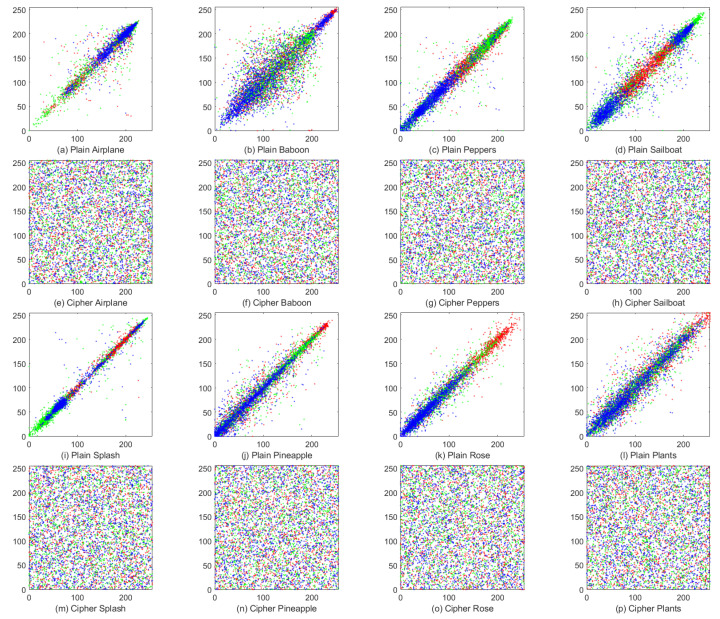
Correlations of 2000 random pairs of adjacent pixels in the horizontal direction. The R, G, and B values of the pixels are shown in red, green, and blue, respectively.

Moreover, we choose 2000 random pairs of adjacent pixels from each plain color image and its corresponding cipher image generated using our presented EDPD to plot the correlations in the horizontal direction in Figure 7. It can be clearly seen that the gray-scale levels of most neighboring pixels of the plain color images are concentrated near the diagonal line, indicating that the high correlations are in these plain color images. In contrast, all the corresponding cipher color images make their pixels fill in the whole planes, showing their quite low correlations. It further confirms that the proposed EDPD has a good ability to break the correlations existing in the plain color images.

According to the above statistical analysis, the cipher color images produced using our proposed EDPD have high information entropies, uniform and flat histograms, and low correlations. These features indicate that the proposed EDPD can effectively resist statistical attacks.

### 4.4. Analysis of Resisting Differential Attacks

Differential attacks are another type of attack that attackers use to compare the variations between a plain image and its cipher image to find the plaintext or desired security key. If a small variation in a plain image causes its corresponding cipher image to produce only a small variation as well, attackers are able to easily crack the encryption scheme. Therefore, a robust image encryption scheme should ensure that even minor modifications in a plain image generate substantially different cipher images, thus fortifying its resistance against differential attacks. There are two common evaluation indicators to measure the ability of the image encryption scheme to resist differential attacks, which are the number of pixels change rate (NPCR) and the unified average changing intensity (UACI). The two indicators are defined by Equation (10) and Equation (11), respectively.

(10) $$NPCR=\frac{1}{h\times w}\sum_{i=1}^{h}\sum_{j=1}^{w}\delta_{i,j}\times 100\%$$

(11) $$UACI=\frac{1}{h\times w\times 255}\sum_{i=1}^{h}\sum_{j=1}^{w}\left|Q_{i,j}^{1}-Q_{i,j}^{2}\right|\times 100\%$$

where *h* and *w* are the height and width of the cipher images $Q^{1}$ and $Q^{2}$; $Q_{i,j}^{1}$ and $Q_{i,j}^{2}$ denote the pixel values at the position of (i,j) in $Q^{1}$ and $Q^{2}$, respectively; and $\delta_{i,j}$ is used to determine whether the pixel values of $Q_{i,j}^{1}$ and $Q_{i,j}^{2}$ differ from each other, as defined and indicated by Equation (12).

(12) $$\delta_{i,j}=\left\{\begin{array}{cc} 0, & Q_{i,j}^{1}=Q_{i,j}^{2}\\ 1, & Q_{i,j}^{1}\neq Q_{i,j}^{2} \end{array}\right.$$

The NPCR denotes the variation ratio of two cipher images, whose plain images are only marginally different. The UACI indicates the average intensity of the differences between two cipher images caused by tiny changes in a plain image. According to the study of Wu et al., given a significance level α = 0.05 and an eight-bit gray-scale image with size of 512 × 512, passing the NPCR test should ensure that the NPCR score exceeds 99.5893%, while passing the UACI test should make the UACI score fall into the range of (33.3730% and 33.5541%) [57]. We add 1 to the value of a randomly selected pixel in a channel of the plain color image and encrypt it, and then compute the NPCR score and the UACI score for this channel using the original cipher image and the new cipher image. This process is repeated ten times for each channel of each color image using our proposed EDPD. The average NPCR and UACI scores are then calculated and compared for the testing images. The results are shown in Table 11 and Table 12. In addition, the values that fail the NPCR or UACI test are italicized within these tables.

From these tables, we can find that all the color image encryption methods pass the NPCR test for all testing images. As a contrary, not every scheme passes the UACI test. In Table 12, the encryption scheme of Ref. [34] fails to reach the passing range of the UACI test in 14 out of 32 cases, and the method of Ref. [51] fails the UACI test once as the score is out of the passing range. Moreover, the proposed EDPD performs well in both the NPCR and UACI tests across all testing images. To summarize, our proposed EDPD can effectively resist differential attacks.

### 4.5. Robust Analysis

In practical scenarios, it is inevitable that noise or data loss occurs during the transmission of images, which leads to contamination of the cipher images. In contrast to the resistance to differential attacks, robustness is concerned with the ability to recover from the decryption of contaminated cipher images. Therefore, an effective image encryption scheme should have enough robustness, making it possible to recover the original images from the decryption of contaminated cipher images to some extent.

**Figure 8:**
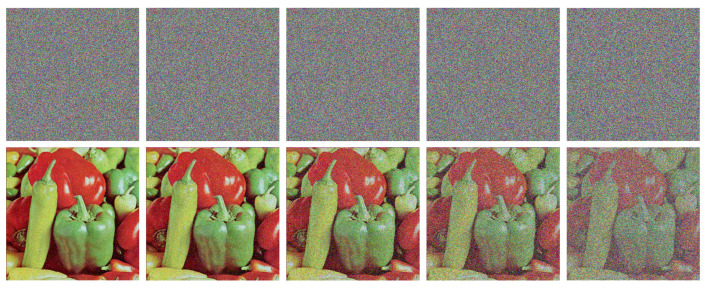
Noise attack results of the cipher image Peppers. The **first row**: cipher images with 0.5%, 1%, 2.5%, 5%, and 10% salt and pepper noise added. The **second row**: the decrypted images from the corresponding cipher images in the **first row**.

To assess the robustness of the proposed EDPD, we first add 0.5%, 1%, 2.5%, 5% and 10% salt and pepper noise to the cipher image Peppers and then decrypt the noisy images using our encryption method. The noise test results are shown in Figure 8, and it is clear that our encryption scheme can recover the cipher images significantly when the noise level is less than 5%. Even in the case of 10% noise, although the decrypted image Peppers is blurry, its profile can be still clearly recognized.

**Figure 9:**
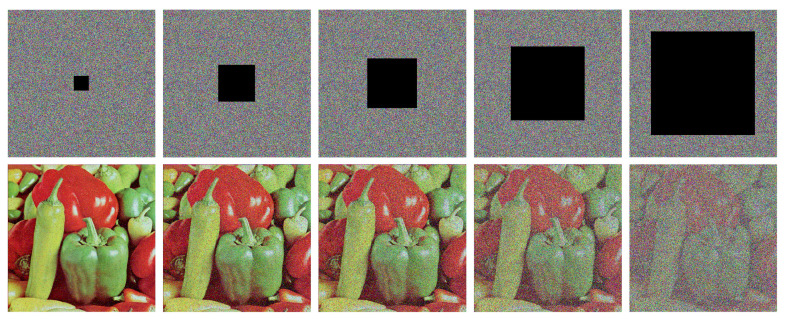
Cropping attack results of the cipher image Peppers (The cropped area is shown in black). The **first row**: cipher images with 1%, 6.25%, 11.3%, 25%, and 50% data loss. The **second row**: the decrypted images from the corresponding cipher images in the **first row**.

Then, we crop 1%, 6.25%, 11.3%, 25%, and 50% pixels at the center of the cipher image Peppers as data loss, and then, use our method to decrypt them and present the crop test results in Figure 9. We can see that, when the data loss ratio is less than 25%, our encryption scheme can recover the image Peppers very well. Even at the highest tested data loss percentage of 50%, the profile and shape of the image Peppers can be also slightly faintly recognized.

In summary, the proposed EDPD has exceptional robustness and can very effectively resist noise attacks and cropping attacks.

### 4.6. Discussion

The proposed EDPD takes about 6.2745 s on average to encrypt a color image of size 512 × 512 × 3 in our experimental environment, while the decryption process takes about 6.1212 s on average. Since the DNA operations are actually string manipulations and the eight-base DNA involves far more encoding rules, our encryption scheme is somewhat time-consuming. An effective way to boost speed is to provide lookup tables for all encoding and algebraic operation rules at the eight-base DNA level.

In hyperchaotic image encryption, many encryption operations depend on generated hyperchaotic sequences that are usually represented by floating-point numbers, which may cause information loss because of their encoding strategies. In the proposed EDPD, we use 64-bit floating-point numbers, which have a significant precision of $10^{-15}$ according to the IEEE 754 standard. Therefore, as long as we retain up to 15 decimal places, we can obtain definite numerical values, resulting in a definite sorting order and integers. Similarly, as long as we use the same floating-point encoding standard and retaining scheme during decryption, we can generate the same hyperchaotic sequence during encryption, enabling us to fully decrypt the image.

The above experimental results indicate that the proposed EDPD is able to resist brute-force attacks, statistical attacks, differential attacks, noise attacks, and cropping attacks. Moreover, since the generation of initial values of the presented four-dimensional hyperchaotic system introduces the influence of the information of the input plaintext color images, every image has its own unique security key to resist known-plaintext attacks and chosen-plaintext attacks. It is worth noting that instead of introducing the plaintext information of color images directly into key generation, we use SHA-512 to extract hash values from plaintext images, split them into multiple parts, and then obtain different keys with some calculations in Section 3.3 of our manuscript, which protects the plaintext information of color images to some extent. In addition, the intricate encryption process and the utilization of hyperchaotic sequences and eight-base DNA computing contribute to the algorithm’s robustness against decryption attempts. Collectively, our proposed EDPD is very effective for image encryption.

The effectiveness of the proposed EDPD owes to its advantages: (1) Information about hash values of the input plaintext color image is introduced into the initial values of the four-dimensional hyperchaotic system to generate the hyperchaotic sequence, which makes each encrypted color image have a unique key and improves the ability of resisting known-plaintext and chosen-plaintext attacks. (2) The eight-base DNA structure brings far more varied encoding rules, and each three-bit block has its own DNA encoding and decoding rules changing with the plain color image. Thus, the diversity of dynamic DNA-level diffusion, as well as the performance and security of color image encryption are improved. (3) The dynamic DNA-level diffusion has the ability to expand a tiny alterations in the input color image to the whole cipher image to resist differential attacks effectively.

## 5. Conclusions

Image encryption is an important task in ensuring the security of images during storage and transmission. Diffusion to change pixel values and permutation to disrupt pixel positions are often common operations for image encryption, and these operations can also be employed on the bit level and DNA level. Furthermore, the manipulations of lower-level data including bits and DNA can affect more pixels and thus improve encryption performance. Bit-level operations generally involve only permutation, while DNA-level operations are suitable for both permutation and diffusion. To enrich the diversity of DNA manipulations, the eight-base DNA-level encoding and algebraic operations are designed according to the widely recognized new eight-base DNA structure. Thus, the proposed EDPD employs the eight-base DNA-level permutation and diffusion, as well as a modified four-dimensional hyperchaotic system.

The proposed EDPD color image encryption approach begins by employing SHA-512 to derive hash information from an input color image and combines it to generate the initial values of the presented hyperchaotic system. Then, it uses the control parameters and initial values of the hyperchaotic system to produce a long enough hyperchaotic sequence, as well as extracts subsequences from it and adjusts the range of values. Finally, two main stages of permutation and diffusion at the one-dimensional sequence level and three-dimensional cube level are designed and constructed as the specific encryption algorithms, which are based on the hyperchaotic subsequences and eight-base DNA encoding with algebraic operations. In the future, innovative approaches to eight-base DNA algebraic operations will be explored, and eight-base DNA-level encryption will be integrated with novel methodologies in conjunction with hyperchaotic systems.

## Figures and Tables

**Table 1:** Traditional DNA encoding rules.

Rule	1	2	3	4	5	6	7	8
00	A	A	C	C	T	T	G	G
01	G	C	A	T	C	G	A	T
10	C	G	T	A	G	C	T	A
11	T	T	G	G	A	A	C	C

**Table 2:** Traditional DNA algebraic addition (⊕), subtraction (⊖), and XOR (⊗) operations.

⊕	A	C	G	T	⊖	A	C	G	T	⊗	A	C	G	T
A	A	C	G	T	A	A	T	G	C	A	A	C	G	T
C	C	G	T	A	C	C	A	T	G	C	C	A	T	G
G	G	T	A	C	G	G	C	A	T	G	G	T	A	C
T	T	A	C	G	T	T	G	C	A	T	T	G	C	A

**Table 3:** Ten of the eight-base DNA encoding rules.

Rule	1	2	3	4	5	6	7	8	9	10
000	A	B	C	Z	T	B	G	S	P	A
001	Z	G	T	A	G	P	B	T	S	B
010	C	A	S	B	P	G	A	P	T	C
011	S	P	Z	C	S	A	Z	C	G	P
100	B	Z	P	G	B	T	P	G	C	Z
101	G	T	B	S	Z	C	T	Z	A	G
110	P	C	A	T	C	Z	S	A	B	S
111	T	S	G	P	A	S	C	B	Z	T

**Table 4:** Eight-base DNA algebraic addition (⊕).

⊕	A	Z	C	S	B	G	P	T
A	A	Z	C	S	B	G	P	T
Z	Z	C	S	B	G	P	T	A
C	C	S	B	G	P	T	A	Z
S	S	B	G	P	T	A	Z	C
B	B	G	P	T	A	Z	C	S
G	G	P	T	A	Z	C	S	B
P	P	T	A	Z	C	S	B	G
T	T	A	Z	C	S	B	G	P

**Table 5:** Eight-base DNA algebraic subtraction (⊖).

⊖	A	Z	C	S	B	G	P	T
A	A	T	P	G	B	S	C	Z
Z	Z	A	T	P	G	B	S	C
C	C	Z	A	T	P	G	B	S
S	S	C	Z	A	T	P	G	B
B	B	S	C	Z	A	T	P	G
G	G	B	S	C	Z	A	T	P
P	P	G	B	S	C	Z	A	T
T	T	P	G	B	S	C	Z	A

**Table 6:** Eight-base DNA algebraic XOR (⊗).

⊗	A	Z	C	S	B	G	P	T
A	A	Z	C	S	B	G	P	T
Z	Z	A	S	C	G	B	T	P
C	C	S	A	Z	P	T	B	G
S	S	C	Z	A	T	P	G	B
B	B	G	P	T	A	Z	C	S
G	G	B	T	P	Z	A	S	C
P	P	T	B	G	C	S	A	Z
T	T	P	G	B	S	C	Z	A

**Table 7:** Experiment parameters.

Parameters Description	Value
User-defined parameters used with SHA-512	$b_{1}=0.1$, $b_{2}=3$, $b_{3}=2\pi$
Control parameters of the hyperchaotic system	a=0.1, b=3, c=2π
Iteration times of the hyperchaotic system	*n* = *h* × *w* × *d* × 2−1
Encryption rounds	R=1

**Table 8:** Test images.

Image	Size (h×w×d)
Airplane	512 × 512 × 3
Baboon	512 × 512 × 3
Peppers	512 × 512 × 3
Sailboat	512 × 512 × 3
Splash	512 × 512 × 3
Pineapple	512 × 512 × 3
Rose	512 × 512 × 3
Plants	512 × 512 × 3

**Table 9:** The IEs of the testing images.

Image	Channel	Input	Cipher Images					
			**EDPD**	**Ref. [10]**	**Ref. [31]**	**Ref. [34]**	**Ref. [36]**	**Ref. [51]**
Airplane	R	6.7178	7.9993	7.9993	7.9993	**7.9994**	7.9993	-
	G	6.7990	**7.9993**	**7.9993**	**7.9993**	**7.9993**	**7.9993**	-
	B	6.2138	**7.9994**	79993	7.9992	7.9993	7.9992	-
Baboon	R	7.7067	7.9993	7.9993	7.9992	**7.9994**	7.9994	-
	G	7.4744	7.9992	7.9993	**7.9995**	7.9994	7.9993	-
	B	7.7522	**7.9994**	7.9993	7.9993	7.9992	**7.9994**	-
Peppers	R	7.3388	7.9992	**7.9994**	7.9992	**7.9994**	**7.9994**	-
	G	7.4963	**7.9993**	**7.9993**	**7.9993**	**7.9993**	7.9992	-
	B	7.0583	**7.9994**	7.9993	**7.9994**	7.9993	7.9993	-
Sailboat	R	7.3124	7.9993	7.9993	7.9992	**7.9994**	**7.9994**	-
	G	7.6429	**7.9993**	**7.9993**	**7.9993**	**7.9993**	7.9992	-
	B	7.2136	7.9992	7.9993	7.9993	**7.9994**	**7.9994**	-
Splash	R	6.9481	7.9992	**7.9994**	7.9993	7.9993	**7.9994**	-
	G	6.8845	**7.9994**	7.9993	7.9992	7.9993	7.9993	-
	B	6.1265	**7.9994**	7.9992	7.9993	7.9993	7.9992	-
Pineapple	R	7.7570	7.9992	**7.9993**	**7.9993**	-	**7.9993**	**7.9993**
	G	7.6830	**7.9993**	**7.9993**	**7.9993**	-	**7.9993**	7.9990
	B	7.2726	7.9993	7.9993	7.9993	-	**7.9994**	7.9992
Rose	R	7.7334	7.9992	7.9992	7.9992	-	**7.9993**	7.9991
	G	7.2488	**7.9993**	**7.9993**	**7.9993**	-	7.9992	**7.9993**
	B	6.8987	**7.9994**	7.9993	7.9992	-	7.9992	7.9989
Plants	R	7.8729	**7.9993**	**7.9993**	**7.9993**	-	**7.9993**	7.9992
	G	7.7675	7.9992	7.9992	**7.9993**	-	**7.9993**	7.9990
	B	7.7106	**7.9993**	**7.9993**	**7.9993**	-	7.9992	7.9991

**Table 10:** The correlation coefficients γ of the testing images.

Image	Channel	γ	Input	Cipher Images					
				**EDPD**	**Ref. [10]**	**Ref. [31]**	**Ref. [34]**	**Ref. [36]**	**Ref. [51]**
Airplane	R	$\gamma_{h}$	0.9726	−0.0010	0.0021	0.0011	−0.0056	**−0.0002**	-
		$\gamma_{v}$	0.9507	−0.0006	−0.0012	**−0.0000**	−0.0151	0.0014	-
		$\gamma_{d}$	0.9346	0.0006	−0.0011	−0.0027	0.0014	**−0.0002**	-
	G	$\gamma_{h}$	0.9425	**−0.0008**	0.0018	−0.0050	−0.0095	−0.0023	-
		$\gamma_{v}$	0.9665	0.0021	−0.0018	0.0015	0.0133	**−0.0010**	-
		$\gamma_{d}$	0.9312	0.0014	0.0026	−0.0012	0.0189	**0.0001**	-
	B	$\gamma_{h}$	0.9633	−0.0012	0.0020	**0.0011**	−0.0025	0.0021	-
		$\gamma_{v}$	0.9162	0.0003	**0.0001**	0.0037	−0.0159	−0.0035	-
		$\gamma_{d}$	0.9110	−0.0002	−0.0020	−0.0003	**−0.0001**	−0.0015	-
Baboon	R	$\gamma_{h}$	0.9218	**−0.0002**	0.0025	0.0033	0.0018	0.0035	-
		$\gamma_{v}$	0.8624	**0.0010**	0.0020	−0.0013	0.0038	0.0013	-
		$\gamma_{d}$	0.8531	−0.0001	**0.0000**	−0.0009	−0.0016	−0.0006	-
	G	$\gamma_{h}$	0.8643	**−0.0008**	−0.0018	0.0018	−0.0013	−0.0013	-
		$\gamma_{v}$	0.7591	−0.0017	−0.0005	**0.0004**	0.0176	0.0011	-
		$\gamma_{d}$	0.7299	−0.0016	0.0015	−0.0003	0.0040	**−0.0001**	-
	B	$\gamma_{h}$	0.9071	0.0017	−0.0019	**−0.0005**	−0.0112	−0.0023	-
		$\gamma_{v}$	0.8782	0.0010	0.0024	**−0.0004**	0.0018	0.0005	-
		$\gamma_{d}$	0.8411	0.0016	0.0013	**−0.0005**	0.0082	−0.0031	-
Peppers	R	$\gamma_{h}$	0.9618	0.0008	0.0019	**0.0002**	0.0054	0.0003	-
		$\gamma_{v}$	0.9640	0.0013	**0.0005**	−0.0011	−0.0042	−0.0009	-
		$\gamma_{d}$	0.9575	0.0037	−0.0024	−0.0024	−0.0177	**−0.0001**	-
	G	$\gamma_{h}$	0.9777	0.0028	0.0027	**−0.0003**	−0.0055	−0.0038	-
		$\gamma_{v}$	0.9771	**0.0021**	0.0035	0.0044	0.0119	−0.0028	-
		$\gamma_{d}$	0.9698	0.0007	−0.0008	**−0.0003**	0.0046	−0.0010	-
	B	$\gamma_{h}$	0.9628	−0.0015	0.0014	0.0034	−0.0021	**0.0009**	-
		$\gamma_{v}$	0.9619	**0.0002**	0.0017	0.0007	0.0104	−0.0062	-
		$\gamma_{d}$	0.9478	−0.0007	**0.0003**	**−0.0003**	−0.0021	−0.0012	-
Sailboat	R	$\gamma_{h}$	0.9544	**−0.0001**	0.0021	−0.0006	−0.0025	−0.0015	-
		$\gamma_{v}$	0.9529	**−0.0005**	−0.0048	0.0011	−0.0092	0.0008	-
		$\gamma_{d}$	0.9396	**−0.0000**	−0.0024	−0.0023	−0.0095	0.0010	-
	G	$\gamma_{h}$	0.9692	−0.0042	0.0023	0.0006	0.0124	**0.0001**	-
		$\gamma_{v}$	0.9627	**0.0008**	−0.0012	0.0013	0.0102	−0.0025	-
		$\gamma_{d}$	0.9520	−0.0011	−0.0013	**−0.0003**	−0.0057	−0.0023	-
	B	$\gamma_{h}$	0.9690	0.0007	0.0032	**−0.0001**	−0.0073	−0.0027	-
		$\gamma_{v}$	0.9688	0.0022	**−0.0016**	0.0019	0.0135	0.0017	-
		$\gamma_{d}$	0.9521	−0.0025	−0.0006	**0.0001**	0.0034	0.0017	-
Splash	R	$\gamma_{h}$	0.9936	0.0026	−0.0029	0.0021	−0.0073	**0.0018**	-
		$\gamma_{v}$	0.9946	**−0.0001**	−0.0028	−0.0012	0.0136	0.0024	-
		$\gamma_{d}$	0.9893	−0.0011	**0.0004**	−0.0007	−0.0006	−0.0023	-
	G	$\gamma_{h}$	0.9796	**−0.0000**	−0.0025	0.0025	−0.0066	−0.0027	-
		$\gamma_{v}$	0.9831	0.0019	0.0008	**−0.0003**	0.0202	0.0017	-
		$\gamma_{d}$	0.9712	−0.0005	0.0009	**0.0002**	−0.0075	−0.0014	-
	B	$\gamma_{h}$	0.9626	0.0038	0.0038	0.0030	−0.0026	**−0.0016**	-
		$\gamma_{v}$	0.9700	**0.0013**	−0.0037	−0.0025	0.0023	0.0024	-
		$\gamma_{d}$	0.9653	**−0.0004**	0.0013	−0.0011	−0.0045	0.0005	-
Pineapple	R	$\gamma_{h}$	0.9819	0.0010	**0.0002**	0.0029	-	−0.0045	**0.0002**
		$\gamma_{v}$	0.9807	0.0024	−0.0031	0.0035	-	**−0.0001**	0.0045
		$\gamma_{d}$	0.9681	−0.0007	0.0005	**0.0003**	-	−0.0012	0.0051
	G	$\gamma_{h}$	0.9753	0.0006	**0.0005**	−0.0024	-	−0.0023	0.0026
		$\gamma_{v}$	0.9739	**−0.0008**	−0.0026	−0.0020	-	−0.0023	0.0012
		$\gamma_{d}$	0.9568	**−0.0001**	−0.0019	−0.0034	-	−0.0015	0.0044
	B	$\gamma_{h}$	0.9586	0.0010	**0.0001**	0.0008	-	−0.0016	0.0037
		$\gamma_{v}$	0.9570	−0.0007	0.0018	0.0023	-	**−0.0000**	0.0046
		$\gamma_{d}$	0.9294	**−0.0004**	−0.0011	0.0009	-	−0.0013	0.0029
Rose	R	$\gamma_{h}$	0.9819	−0.0035	**−0.0018**	−0.0039	-	0.0048	0.0028
		$\gamma_{v}$	0.9831	−0.0007	−0.0016	**0.0002**	-	−0.0019	0.0049
		$\gamma_{d}$	0.9703	−0.0008	**−0.0001**	−0.0002	-	0.0008	0.0049
	G	$\gamma_{h}$	0.9646	0.0026	−0.0031	0.0015	-	**−0.0001**	0.0055
		$\gamma_{v}$	0.9641	0.0040	0.0005	0.0009	-	**−0.0003**	0.0023
		$\gamma_{d}$	0.9371	0.0033	**0.0000**	−0.0018	-	−0.0012	0.0086
	B	$\gamma_{h}$	0.9458	−0.0034	0.0004	−0.0018	-	**−0.0002**	0.0034
		$\gamma_{v}$	0.9449	−0.0016	**0.0001**	0.0027	-	0.0005	0.0053
		$\gamma_{d}$	0.9026	**0.0001**	0.0010	**−0.0001**	-	−0.0005	0.0014
Plants	R	$\gamma_{h}$	0.9545	−0.0002	0.0007	**0.0001**	-	0.0024	0.0010
		$\gamma_{v}$	0.9581	−0.0007	**−0.0003**	0.0008	-	−0.0015	0.0081
		$\gamma_{d}$	0.9249	0.0022	−0.0007	0.0038	-	−0.0005	0.0004
	G	$\gamma_{h}$	0.9488	**−0.0000**	−0.0017	−0.0030	-	0.0007	0.0052
		$\gamma_{v}$	0.9523	**0.0002**	0.0023	−0.0029	-	0.0006	0.0043
		$\gamma_{d}$	0.9151	0.0024	**−0.0010**	0.0015	-	0.0017	0.0025
	B	$\gamma_{h}$	0.9484	−0.0011	−0.0010	0.0034	-	**0.0004**	0.0057
		$\gamma_{v}$	0.9519	−0.0026	0.0015	**0.0005**	-	0.0046	0.0042
		$\gamma_{d}$	0.9143	**−0.0011**	**−0.0011**	−0.0014	-	−0.0012	0.0033

**Table 11:** The NPCR (%) of the testing images.

Image	Channel	Cipher Images					
		**EDPD**	**Ref. [10]**	**Ref. [31]**	**Ref. [34]**	**Ref. [36]**	**Ref. [51]**
Airplane	R	99.6056	99.6118	99.6045	99.6063	99.6121	-
	G	99.6116	99.6107	99.6125	99.6033	99.6164	-
	B	99.6138	99.6184	99.6233	99.6029	99.6107	-
	Average	99.6103	99.6136	99.6134	99.6042	99.6131	-
Baboon	R	99.6045	99.6117	99.6029	99.6048	99.6104	-
	G	99.6108	99.6109	99.6069	99.6059	99.6106	-
	B	99.6191	99.6094	99.6117	99.6071	99.6090	-
	Average	99.6115	99.6107	99.6072	99.6059	99.6100	-
Peppers	R	99.6080	99.6074	99.6144	99.5975	99.6118	-
	G	99.6069	99.6064	99.6177	99.6052	99.6047	-
	B	99.6041	99.6048	99.6190	99.6037	99.6122	-
	Average	99.6063	99.6062	99.6170	99.6021	99.6096	-
Sailboat	R	99.6112	99.6144	99.6045	99.6089	99.6058	-
	G	99.6070	99.6069	99.6167	99.5983	99.6047	-
	B	99.6061	99.6117	99.5990	99.6174	99.6035	-
	Average	99.6081	99.6110	99.6067	99.6082	99.6047	-
Splash	R	99.6087	99.6118	99.6135	99.6037	99.6064	-
	G	99.6112	99.6114	99.6110	99.6020	99.6084	-
	B	99.6050	99.6078	99.5993	99.6082	99.6080	-
	Average	99.6083	99.6103	99.6079	99.6046	99.6076	-
Pineapple	R	99.6039	99.6091	99.5932	-	99.6123	-
	G	99.6019	99.6014	99.6043	-	99.6093	-
	B	99.6093	99.6034	99.6029	-	99.6085	-
	Average	99.6050	99.6046	99.6001	-	99.6100	99.6138
Rose	R	99.6017	99.6128	99.6126	-	99.6169	-
	G	99.6138	99.6098	99.6112	-	99.6048	-
	B	99.6049	99.6069	99.6080	-	99.6028	-
	Average	99.6068	99.6098	99.6106	-	99.6082	99.6204
Plants	R	99.6007	99.6013	99.6052	-	99.6074	-
	G	99.6059	99.6136	99.6087	-	99.6024	-
	B	99.6132	99.6062	99.6064	-	99.6111	-
	Average	99.6066	99.6070	99.6067	-	99.6070	99.6097

**Table 12:** The UACI (%) of the testing images.

Image	Channel	Cipher Images					
		**EDPD**	**Ref. [10]**	**Ref. [31]**	**Ref. [34]**	**Ref. [36]**	**Ref. [51]**
Airplane	R	33.4938	33.4655	33.4393	*31.9665*	33.4671	-
	G	33.4756	33.4599	33.4644	*33.1449*	33.4304	-
	B	33.4568	33.4647	33.4332	*32.7264*	33.4561	-
	Average	33.4754	33.4634	33.4456	*32.6126*	33.4512	-
Baboon	R	33.4655	33.4583	33.4285	*29.9931*	33.4538	-
	G	33.4101	33.4907	33.4941	*28.5822*	33.4838	-
	B	33.4822	33.4578	33.4867	*31.2384*	33.4615	-
	Average	33.4526	33.4689	33.4698	*29.9379*	33.4664	-
Peppers	R	33.4564	33.4537	33.4579	*29.0588*	33.4929	-
	G	33.4727	33.4811	33.4696	33.4382	33.4243	-
	B	33.4897	33.3860	33.4905	33.4001	33.4763	-
	Average	33.4729	33.4402	33.4727	*31.9657*	33.4645	-
Sailboat	R	33.4386	33.4532	33.4763	*27.9264*	33.4981	-
	G	33.4555	33.4721	33.4765	33.4203	33.4675	-
	B	33.4438	33.4793	33.4591	33.4025	33.4551	-
	Average	33.4460	33.4682	33.4706	*31.5831*	33.4736	-
Splash	R	33.5129	33.4724	33.4592	33.4427	33.4524	-
	G	33.4478	33.4659	33.4771	33.4605	33.4739	-
	B	33.4273	33.4741	33.4828	*31.9747*	33.4991	-
	Average	33.4627	33.4708	33.4730	*32.9593*	33.4751	-
Pineapple	R	33.4567	33.4814	33.4675	-	33.4670	-
	G	33.4468	33.4633	33.4837	-	33.4479	-
	B	33.4682	33.4748	33.4635	-	33.4832	-
	Average	33.4572	33.4732	33.4716	-	33.4660	33.4944
Rose	R	33.4883	33.4874	33.4716	-	33.4700	-
	G	33.4388	33.4603	33.4548	-	33.5072	-
	B	33.4678	33.4481	33.4917	-	33.4819	-
	Average	33.4650	33.4653	33.4727	-	33.4864	33.5147
Plants	R	33.4920	33.4548	33.4880	-	33.4735	-
	G	33.4955	33.4689	33.4253	-	33.4004	-
	B	33.4887	33.4661	33.4734	-	33.4659	-
	Average	33.4921	33.4633	33.4622	-	33.4466	*33.5643*

## Data Availability

The used testing images are all included in the paper.
